# A Technology for Developing Synbodies with Antibacterial Activity

**DOI:** 10.1371/journal.pone.0054162

**Published:** 2013-01-23

**Authors:** Valeriy Domenyuk, Andrey Loskutov, Stephen Albert Johnston, Chris W. Diehnelt

**Affiliations:** 1 The Biodesign Institute of Arizona State University, Tempe, Arizona, United States of America; 2 School of Life Science, Arizona State University, Tempe, Arizona, United States of America; Institut Pasteur, France

## Abstract

The rise in antibiotic resistance has led to an increased research focus on discovery of new antibacterial candidates. While broad-spectrum antibiotics are widely pursued, there is evidence that resistance arises in part from the wide spread use of these antibiotics. Our group has developed a system to produce protein affinity agents, called synbodies, which have high affinity and specificity for their target. In this report, we describe the adaptation of this system to produce new antibacterial candidates towards a target bacterium. The system functions by screening target bacteria against an array of 10,000 random sequence peptides and, using a combination of membrane labeling and intracellular dyes, we identified peptides with target specific binding or killing functions. Binding and lytic peptides were identified in this manner and *in vitro* tests confirmed the activity of the lead peptides. A peptide with antibacterial activity was linked to a peptide specifically binding *Staphylococcus aureus* to create a synbody with increased antibacterial activity. Subsequent tests showed that this peptide could block *S. aureus* induced killing of HEK293 cells in a co-culture experiment. These results demonstrate the feasibility of using the synbody system to discover new antibacterial candidate agents.

## Introduction

While there is no perfect understanding of the forces directing evolution of antibiotic resistance a prominent view holds that these issues are in part the consequence of the widespread use of broad spectrum antibiotics [1], [2]. It has been proposed that next generation antimicrobial treatments must focus on: developing pathogen-specific antibiotics, greatly improving diagnostics, and expanding the role of immunotherapy [3]. Along these lines, there has been a resurgence of monoclonal antibody (mAbs) based therapeutic development [4], [5]. Historically, antibody therapies were the first effective anti-infective agents (e.g. for pneumonia, meningitis, erysipelas). However, their wide usage is restricted by the high cost of development and production of pathogen specific mAbs and the large number of current antimicrobial drugs on the market. Other groups are developing antimicrobial peptides (APs) as a means of avoiding resistance, as there have been few reports of resistance arising to APs [6]. Despite numerous attempts to develop new AP-based therapeutics using either natural [7], [8], optimized via amino acid substitutions [9]–[12] or dimeric peptides [13], [14], only a few products have reached the market [15]. APs have two limitations. One is that there are relatively few for development [16] (131 APs for Gram-negative bacteria and 283 for Gram-positive peptides in Antimicrobial Peptide Database, February 2012). The second is that they generally have high toxicity and broad activity, which is consistent with their evolutionary origin [17].

Our group has previously developed a class of affinity agents called synbodies that are produced by screening the target of interest against a peptide microarray to discover low affinity peptides that are then joined on a scaffold to produce high affinity, highly specific binding agents [18]–[20]. Synbodies can be easily modified to increase affinity [20], [21], have an orthogonal functional group that can be used for conjugation to a wide variety of moieties, and should be ideal lead therapeutic candidates. We sought to extend this platform to bacteria in an attempt to create synbodies with specificity towards a target pathogen that can function as new antibacterial candidates. By targeting the bacterial surface, we should reduce the likelihood of the target bacteria developing resistance.

**Figure 1:**
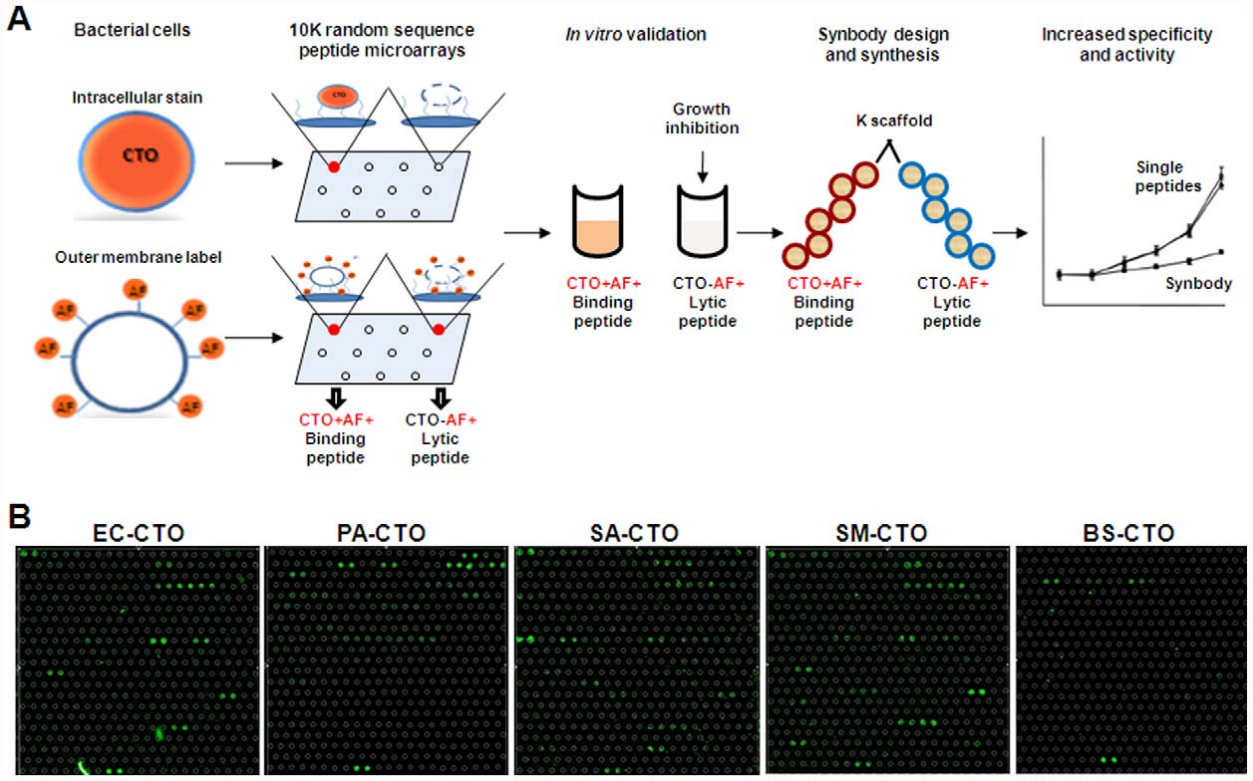
Bacteria binding to peptide microarrays. (**A**) Workflow to develop pathogen specific antibiotics. Bacterial cells are applied to the peptide microarray carrying dyes either in cytoplasm or on the membrane. Intracellular dye Cell Tracker Orange (CTO) identifies peptides that bind bacterial cells without disrupting the cell membrane while the outer membrane label Alexa Fluor 555 identifies either intact or dead cells. Comparing the profiles of a pathogen at the same peptide sequence enables the selection of peptides with binding or lytic activity. After *in vitro* validation, linking together a peptide with antimicrobial activity and a specific binder for the pathogen produces a synbody. (**B**) Distinct profiles of CTO stained *E.coli* O111:B4 (EC), *P. aeruginosa* (PA), *S. aureus* (SA), *S. mutans* (SM), *B. subtilis* (BS) on representative sub-array (48 peptides from 10,000 total). Cell binding signals are depicted as a false color (green).

The discovery platform is similar to the platform we have used to develop synbodies to protein targets but with a few important modifications: 1) whole bacteria are screened against the 10,000 random-sequence peptides microarray; 2) pathogen specific peptides with binding or lytic action are identified from a microarray functional screening assay and 3) combining binding and lytic peptides produces synbodies with activity against a particular pathogen (Figure 1A). By screening whole bacteria, we have the ability to profile any possible pathogen without selecting specific surface components, such as lipoteichoic acids, proteins and peptidoglycans for Gram-positive bacteria or lipopolysaccharides (LPS) for Gram-negative bacteria. The microarray based functional assay distinguishes between peptides with antimicrobial activity and those that bind without affecting growth providing a large source of pathogen specific peptides that can be identified in a rapid manner. We hypothesize that this technology for developing specific antibiotics to any pathogen will provide a straightforward method for designing new compounds. As a test of this system, we developed a synbody against *Staphylococcus aureus* (SA), the Gram-positive bacteria that is one of the causative agents of hospital acquired bacterial pneumonia [22] to demonstrate a general method to create new antibacterial candidates.

## Results

### Bacterial Cell Binding to Peptide Microarrays

**Figure 2:**
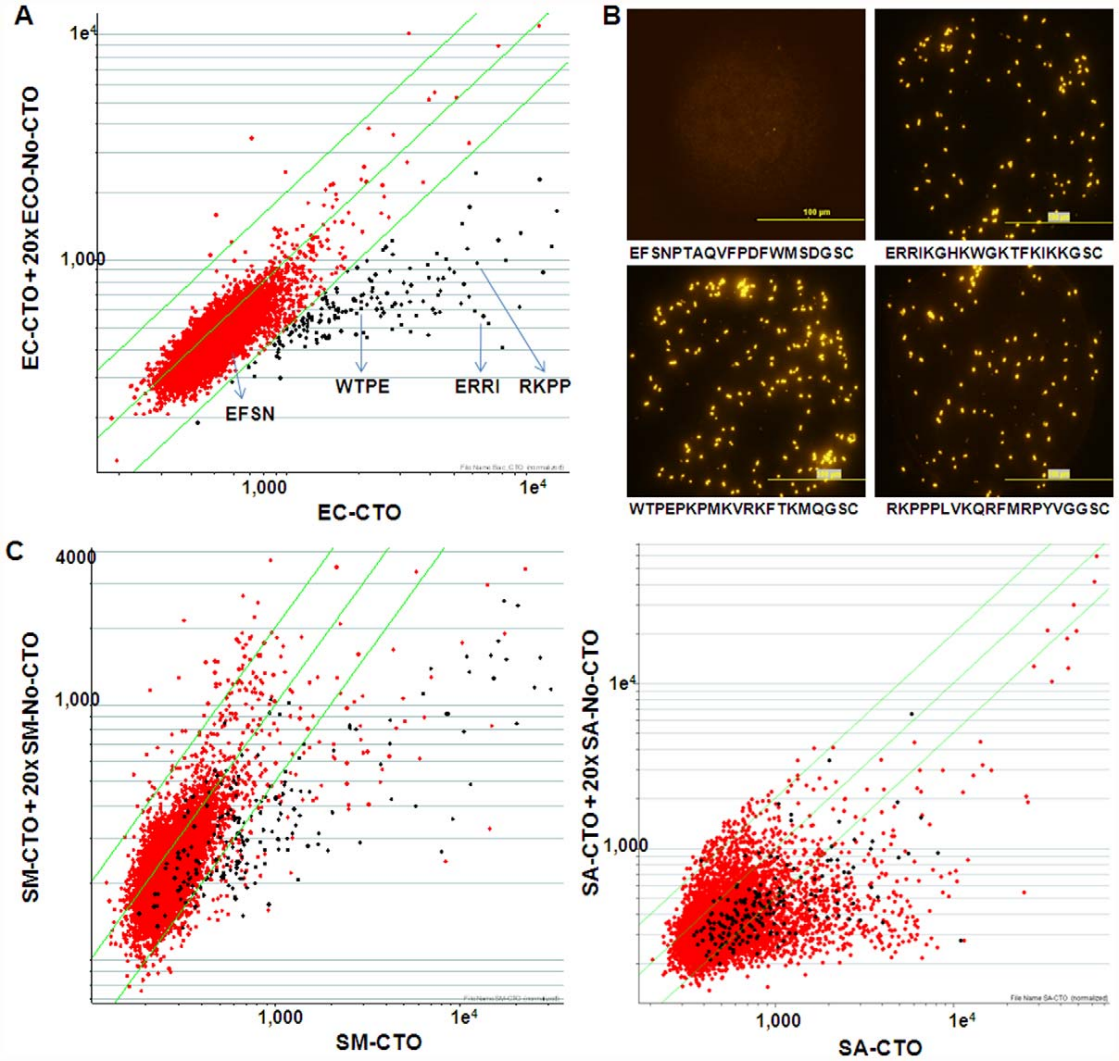
Demonstration of bacteria binding to peptide microarrays. (**A**) Binding data of CTO stained EC (x axes) plotted vs negative control (y axes). Both axes show raw median fluorescent signal at 543 nm on a logarithmic scale. Green lines delimit the twofold change. Dark dots outside of two-fold change are binder-candidates. (**B**) EC binding to peptides annotated in (A) on custom polymer microarray detected by fluorescent microscopy. Upper left image is negative control (non-binding peptide EFSN). Scale – 100 µm. (**C**) Binding and competition dataset for SM and SA. Annotated dark dots are peptide-binders for EC selected in (A) and demonstrate binding specificity.

The basic elements of the synbody process are shown in Figure 1A. In the first step, labeled bacteria are applied to the peptide microarray, which consists of 10,000 peptides of 20aa length. The composition of each peptide is known, but generated by a random number generator using 19 aa (cysteine was omitted). We found that the (3-aminopropyl)triethoxysilane (APTES) microarray surface chemistry used in earlier published reports of screening proteins, antibodies and carbohydrates [18], [23]–[25] showed high levels of non-specific binding to the interstitial regions of the array and low level binding to peptide spots when whole bacterial cells were screened. To address these issues we developed an alternative microarray surface chemistry using a hyperbranched polymer that reduced non-specific binding and increased the peptide density (Figure S1). We chose a diverse set of bacteria to test the general applicability of this approach and screened Gram-negative bacteria, *Escherichia coli* O111:B4 (EC) and *Pseudomonas aeruginosa* (PA); and Gram-positive bacteria, *Streptococcus mutans* (SM), *Staphylococcus aureus* (SA) and *Bacillus subtilis* (BS) against the 10,000 peptide library. In order to identify peptides that bound to the bacteria surface but did not affect the function of the bacterial membrane, we used Cell Tracker Orange (CTO), an internalizing dye that is activated upon entering bacterial cells and remains inside intact cells. Each bacterium was labeled with CTO and screened against the peptide microarray with each experiment performed in duplicate at with four technical replicates per peptide sequence. The raw images represent the same area of five peptide microarrays processed with different bacterial species (Figure 1B), which bind in a clearly distinguished pattern. A competition experiment was also performed in which the CTO labeled bacterium was screened in the presence of 20× excess of un-labeled bacteria to confirm the bacterial cell binding. Construction of scatter plots [25], [26] for both datasets (binding and competition) (Figure 2A) reveal peptides with signals that are at least twofold higher than those in the presence of excess un-labeled bacteria (black filled circles in Figure 2A). Whole bacterial cell binding can clearly be seen in Figure 2B, where EC binding to selected peptides (highlighted in Figure 2A) was visualized by fluorescent microscopy. This assay is a straightforward method to identify peptides that specifically bind a pathogen and works well for both Gram-negative and Gram-positive bacteria (Figure 2A,C).

### Identification of Antimicrobial Peptides on a Peptide Microarray

**Figure 3:**
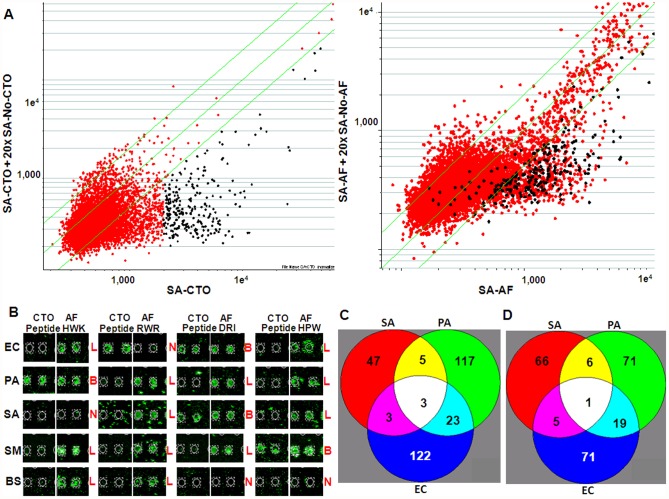
Selection of binding and lytic peptides from microarray screening. (**A**) Scatter plots comparing binding/competition data for SA with intracellular stain CTO (left) and membrane label AF (right). Both axes show raw median fluorescent signal at 543 nm on a logarithmic scale. Green lines delimit the twofold change. Peptide-binders (dark dots) are selected out of twofold change on x axes as those where CTO-cells were competed with excess of non-stained cells and repeated with AF labeled cells. Other peptides out of twofold change on x axes at SA-AF (red dots) are considered lytic as they were not detected with CTO. (**B**) Microarray binding of CTO stained vs AF labeled EC, PA, SA, SM and BS for peptides HWK, RWR, DRI, HPW (spotted in duplicate). L = lytic peptide (CTO-AF+). B = binding peptide (CTO+AF+). N = no binding. (**C**) Specificity and uniqueness of bacterial profiles at peptide microarray in binding peptides dataset (**D**) and for lytic peptides dataset. SA data presented in Venn diagrams have had SM and BS binding peptides filtered out.

While this technique works well to select peptides that specifically bind surface components on the target bacteria, these peptides might not have intrinsic antibacterial activity. As depicted in Figure 1, this concept of the synbody involves combining a high specificity binding peptide with a peptide that has high antibacterial activity, such as a naturally occurring antimicrobial peptide. However, many naturally occurring APs function by disruption of the bacterial membrane and have been evolutionarily optimized to have broad activity and can be quite toxic [17]. We hypothesized that we could use the peptide microarray to identify both binding peptides and lytic peptides. To do so we used an N-hydroxysuccinimide (NHS) ester activated dye, AlexaFluor-555 (AF) to label the primary amines present in the bacterial membrane. Peptides that bind but do not disrupt the bacterial membrane should have signal when detected with both CTO and AF (CTO+AF+) while peptides with lytic activity cause CTO to leak out of cells once the membrane is disrupted (CTO-AF+). The binding/competition assay was performed with SA labeled with both dyes and peptides that showed binding to SA-CTO (black circles) were inspected on the scatter plot with AF data (Figure 3A). Most of the peptides repeated in the AF data can be considered as binder-candidates (CTO+AF+). However, there are additional peptides (red circles) in this area that may also have lytic activity (CTO-AF+). Examples of functional assay performance with other bacteria EC, PA, BS, SM can be seen in Supplementary Figure S2. Examples of microarray profiles of CTO and AF labeled bacteria for four different peptides show that the profile of binding peptides and lytic peptides can be clearly distinguished (Figure 3B). We then analyzed this data to determine the specificity of binding and lytic peptides across the five bacteria tested. Using a linear classifier, we were able to identify specific binding (Figure 3C) as well as lytic (Figure 3D) peptide-candidates for SA.

### Validation of *in vitro* Activity of Microarray Selected Peptides

**Figure 4:**
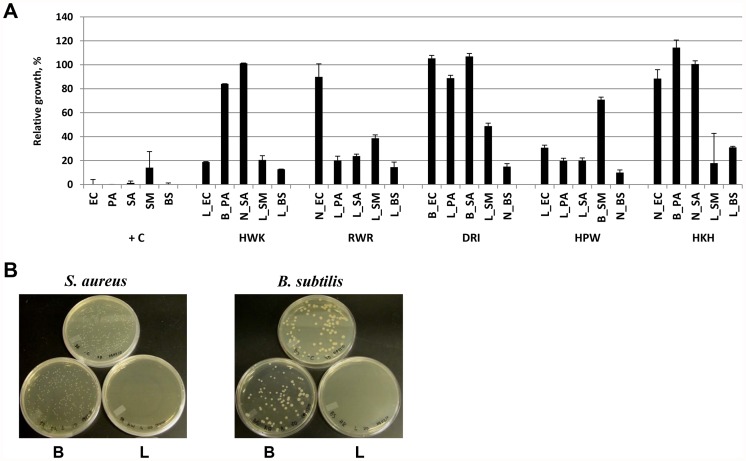
Validation of microarray predicted lytic activities of peptides. (**A**) Relative growth inhibition of EC, PA, SA, SM and BS by peptides HWK, RWR, DRI, HPW, HKH at 100 µM. End-point measurement after 18 h. “+ C” - positive control kanamycin, 100 µM. L – microarray predicted lytic activity of peptide B – binding activity of peptide. N – no microarray profile. The error bars are standard deviations of triplicate measurements. (**B**) Cultures were plated after 5 minute incubation with 100 µM of binding or lytic peptides. Pictures were taken after 24 hours growth. Upper plate is negative control for each strain. Peptide-binders: DRI for SA; KQK for BS. Lytic peptides: RWR for SA; HRK for BS.

We then tested the correlation between peptides with microarray predicted activity and their ability to inhibit bacterial growth *in vitro*. Based on their microarray profile, we chose 40 peptides that were identified as binding peptides, lytic peptides, or non-binding peptides for EC, PA, SA, SM, and BS and tested each peptide in a standard bacterial growth inhibition assay. In this manner, we could assess the performance of the selection process. Peptides were screened at 100 µM (Figure 4A) and the number of peptides that inhibited bacterial growth greater than 50% is shown in Table 1 along with the average inhibition of each group. As can be seen, more than 50% of peptides that were predicted to have lytic activity from the microarray assay had inhibitory activity *in vitro* and those peptides showed stronger growth inhibition compared to either peptide binders or non-binding peptides. The correlation between inhibitory activity of immobilized peptides and their behavior in solution was quite good considering that the density of peptide immobilized on a microarray is quite high. It is possible that the high avidity of the peptide on the microarray causes some peptides to have a lytic phenotype when immobilized but not have activity when free in solution. We next determined the minimum inhibitory concentration (MIC) for a subset of peptides that showed growth inhibition (Table S1). From these data, it can be seen that a number of peptides showed broad bacterial inhibition while other peptides had little to no activity. Microarray predicted properties of peptides were additionally confirmed by growing bacterial cultures on the plates after mixing with lytic or binding peptides (Figure 4B). Peptides that were predicted to be lytic from the microarray screen inhibited bacteria growth while binding peptides did not. Based on these results, the microarray screen does select peptides with antibacterial activity.

**Table 1:** Summary of in vitro growth inhibition screening of microarray selected peptides.

	Actual versus Predicted Inhibition			Average Inhibition		
Bacteria	Peptide Binders	Lytic Peptides	Non-Binders	Peptide Binders	Lytic Peptides	Non-Binders
*E. coli*	0/11	5/11	1/11	0%	76%	50%
*P. aeruginosa*	0/17	6/14	0/7	0	82%	0%
*S. aureus*	0/8	6/8	0/22	0%	84%	0%
*S. mutans*	4/22	8/12	0/4	41%	68%	0%
*B. subtilis*	0/4	13/15	3/19	0%	80%	77%

### Evaluation of a *S. aureus* Synbody

**Figure 5:**
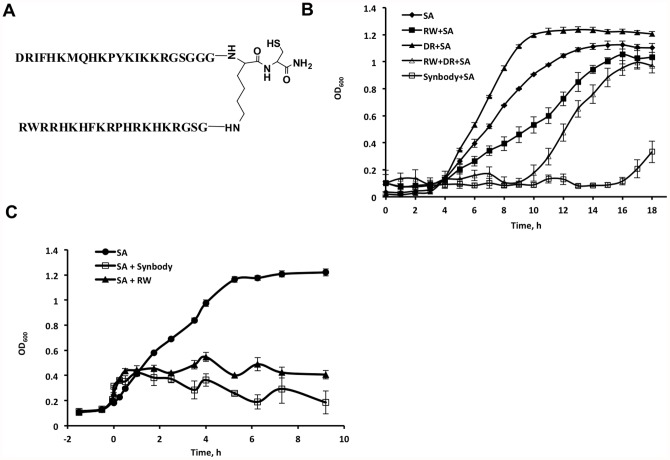
*In vitro* characterization of *S. aureus* synbody. (**A**) Structure of *S. aureus* synbody. (**B**) Bacterial growth of *S. aureus* over time after treatment with peptides or synbody. Measurements were taken hourly. Data points represent the average of three independent experiments. Starting *S. aureus* concentration is ∼2×10^5^ CFU/mL. (**C**) Test of lytic activity of lytic peptide and synbody. *S. aureus* was grown until ∼1.7×10^9^ CFU/mL and the peptide or synbody was added to the final concentration of 100 µM at time zero. Data points represent the average of three independent experiments.

To develop a *S. aureus* synbody, we combined a peptide with activity against SA and a SA specific binding peptide (Figure 5A) similar to the approach described in [27], [28]. Peptide RWRRHKHFKRPHRKHKRGSC (peptide RW) was selected for its activity for SA and PA, while peptide DRIFHKMQHKPYKIKKRGSC (peptide DR) was selected for its high binding to SA on the peptide microarray. This synbody had two times higher antibacterial activity for SA than the original lytic peptide yet did not inhibit growth of *E. coli* or *S. mutans* (Table 2). This was not observed for *P. aeruginosa* where the MIC of the synbody is roughly the same as that of the lytic peptide. While the synbody had activity against *B. subtilis*, this bacterium was the most susceptible to growth inhibition by individual peptides (Table S1). We also tested the antibacterial activity against three species of bacteria that were not included in the selection process: the closely related commensal bacteria *Staphylococcus epidermidis;* and two other Gram-negative bacteria, *Escherichia coli* O157:B7 and *Burkholderia thailandensis*. It was found (Figure S3) that the synbody inhibited the growth of *S. epidermidis* (MIC = 1.75 µM) and *E. coli O157:B7* (MIC = 14 µM) while it did not inhibit the growth of *B. thailandensis*, which is resistant to many antibacterial peptides [29]. Analysis of bacterial growth kinetics (Figure 5B) illustrates the advantage of the synbody over the original peptides at 25 µM concentration of each. Noticeable growth of *S. aureus* in the presence of the peptide RW was detected after 4 hours incubation, while in the presence of synbody noticeable growth was detected only after 16 hours. Interestingly, a mixture (not conjugate) of the two original peptides RW and DR also shows improved activity, with bacterial growth suppressed for 9 hours. However, a mixture of peptides is still less effective than the synbody.

**Table 2:** MIC values of *S. aureus* binding peptide, inhibitory peptide, and synbody.

	Gram-positive Bacteria			Gram-Negative Bacteria	
	*S. aureus*	*S. mutans*	*B. subtilis*	*E. coli* O111:B4	*P. aeruginosa*
DRIFHKMQHKPYKIKKRGSC	*N.I.*	*N.I.*	*N.I.*	*N.I.*	*N.I.*
RWRRHKHFKRPHRKHKRGSC	28±1.5 µM	*N.I.*	*N.I.*	*N.I.*	27±2.3 µM
Synbody	14±0.8 µM	*N.I.*	14±1.1 µM	*N.I.*	22±1.8 µM

*N.I.* = no inhibition.

The lytic activity of the synbody and the peptide RW was tested by adding each to a *S. aureus* culture at ∼1/3 of log phase at ∼1.7×10^9^ CFU/ml (Figure 5C). The growth of *S. aureus* in the presence of the peptide plateaus while bacteria treated with synbody shows a gradual reduction in OD after synbody addition. This suggests a bacteriostatic rather than bactericidal effect of the synbody given that the synbody was added at a concentration that was 7 times the MIC. To further clarify this effect, we performed a kill curve kinetic study of the synbody and each peptide (Table S2). As can be seen, kanamycin has a rapid bactericidal effect (>3 log_10_ reduction in CFU/mL) when added at 2 times and 4 times the MIC. However, neither the synbody nor the individual peptides had a bactericidal effect at 4 times the MIC. Thus, we conclude that the synbody has a bacteriostatic rather than a bactericidal effect on *S. aureus*.

### The Synbody Inhibits *S. aureus* Induced Cell Death

**Figure 6:**
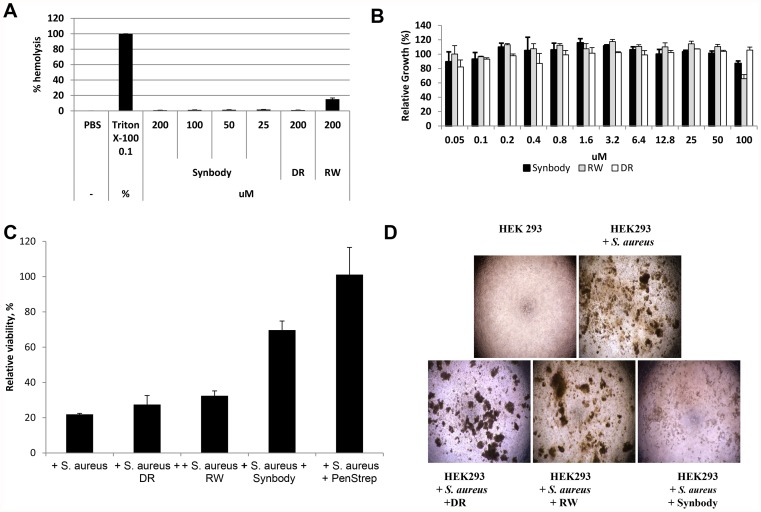
Testing of synbody toxicity and demonstration of a protective effect on human cells in co-culture with *S. aureus*. (**A**) Hemolytic activity of SA synbody and individual peptides on mouse red blood cells. (**B**) Test of synbody cytotoxicity for HEK293 cells versus original peptides. The synbody was added to cells for 48 hours and cell viability was measured by BrdU proliferation assay. (**C**) Viability of HEK293 cells in co-culture with *S. aureus* with and without synbody treatment, as measured by cellular ATP content measurement. Data are normalized to the cellular ATP content of cells only. Synbody (25 µM), peptide (25 µM) or antibiotic control (180 µM) was added to co-culture immediately after mixing. The error bars represent the standard deviation of triplicate measurements. (**D**) Light microscopy (10×) of cells only, cells in co-culture with *S. aureus* for 24 hours, cells treated with 25 µM RW peptide and cells treated with 25 µM synbody.

To ensure that the synbody was not highly toxic like many antimicrobial peptides, we tested the synbody for toxicity against mammalian cells using a standard hemolytic assay against murine red blood cells (Figure 6A) and a growth inhibition assay against human kidney cells (HEK293) (Figure 6B). It was found that the synbody did not lyse mouse red blood cells or inhibit the growth of HEK293 cells. These data demonstrate that we can combine a peptide with rather broad growth inhibition activity with a peptide that specifically binds *S. aureus* to produce a synbody without a widespread toxic effect.

After this preliminary toxicity test, we wished to determine if the synbody could protect mammalian cells from bacterial induced cell death. We used a model system where human embryonic kidney cells (HEK293) were co-cultured with *S. aureus* to simulate *S. aureus* infection of kidney cells in transplants [30]. Cell viability was determined by cellular ATP content and was normalized to 100% for the cells alone control. We tested a range of conditions at which *S. aureus* can grow in tissue culture media and found that there was significant decline in HEK293 viability with as little as 1.25×10^3^ CFU/ml of *S. aureus*. The final experimental setup consisted of 1.25×10^6^ CFU/ml of SA (1000× higher than minimal harmful concentration of bacteria) added alone or with synbody or the individual peptides added to at 25 µM. A 180 µM solution of PenStrep was used as a positive control and provided complete protection in this assay. Treatment with the individual peptides offered no protection from *S. aureus* induced cell death while the synbody treated cells maintained about 70% cell viability (Figure 6C). The protective effect of the synbody on human cells was confirmed using light microscopy after 24 hours of treatment (Figure 6D). Large colonies of *S. aureus* and dead cells are clearly seen in the untreated image while the synbody treated sample has few colonies of *S. aureus* present. These results demonstrate that the synbody has a protective effect against a simulated *S. aureus* infection.

## Discussion

Here we demonstrate a simple and general method for screening bacteria on peptide microarrays. We have shown that a variety of bacteria can be panned and simultaneously screened for peptides that bind the bacteria or lyse them, using a two dye system. When these peptides are resynthesized and tested in solution, over half manifest the phenotype selected on the array. When a SA specific binding peptide was combined in one synbody with a broadly lytic peptide, the specificity and activity was improved. This improved synbody was relatively non-toxic on mammalian cells in culture. Finally, we show that the synbody is also effective in an *in vitro* assay for protecting human cells against *S. aureus*.

The rise of multiple sources of antibiotic resistance creates urgency not only in the development of new antibiotics but on the development of new systems of producing antibiotic candidates. We are focusing on technologies to create species specific antibiotics that would be difficult to develop resistance to. Ideally, this technology could be applied in a systematic method that is both rapid and cost effective. In this work, we have demonstrated the feasibility of such a system to quickly develop an antimicrobial synbody that has a relatively narrow specificity for *Staphylococcus aureus*.

While others have detected antimicrobial activity on cellulose-tethered peptides using bacteria transformed with luciferase gene [31], [32] our method offers several advantages over this method: (i) the functional assay allows detection of lytic peptides as well as peptide-binders directly on microarrays; (ii) the small peptide library was characterized prior to spotting, in contrast to peptides synthesized directly on the cellulose support which are not characterized prior to use; (iii) the hyperbranched polymer surface has higher sensitivity than other surfaces; and finally, (iv) bacteria can be profiled in as little as 2 hours. The unique combination of bacterial profiling on a microarray with subsequent selection of binding versus lytic peptides enables a very straightforward process for selection of pathogen specific candidates. The technology for designing a synbody based on a pathogen's microarray profile that combines two or more peptide characteristics is fundamentally new to the antimicrobial field. The flexibility of the platform suggests that it would be possible to use this system to develop antibacterial candidates to almost any pathogen in a rapid manner. It should also be possible to improve the activity and possibly specificity of the synbody for a target bacterium. As has been demonstrated with proteins [18], [20] combining two or more peptides can produce synbodies with much higher affinity. A simple mutagenesis protocol to improve peptide features for binding proteins has also been presented [21] which may also be applicable to bacteria. While peptide therapeutics have issues with *in vivo* stability and pharmacokinetics, there are numerous advances that have been made in the stabilization of peptide therapeutics, that make the synbody approach a viable source of new antibacterial candidates.

## Materials and Methods

Animal experiments were conducted following an animal use protocol (1099R) which was reviewed and approved by the Arizona State University Institutional Animal Care and Use Committee. Human tissue culture experiments were approved by Arizona State University Institutional Review Board (Protocol # 0508000152).

Microarray peptides were synthesized by Alta Biosciences Ltd. (Birmingham, UK). Lead peptides were synthesized in-house or by Sigma Aldrich by using Fmoc chemistry and purified to 95% by HPLC. Spectrophotometric measurements were carried out by using a NanoDrop® ND-1000, SpectraMax 190 and M5 (Molecular Devices). Microarrays were scanned with ProScanArray HT microarray scanner (Perkin Elmer).

### Bacteria

*Escherichia coli* O111:B4 (ATCC) was grown at 37°C in Difco nutrient broth medium for non-fastidious organisms (Becton, Dickinson and Company 231000). *Bacillus subtilis* 1A423, *Pseudomonas aeruginosa* PAO-1 and *Staphylococcus aureus* UAB637 (kindly provided by Center for Infectious Diseases and Vaccinology (CIDV), the Biodesign Institute at Arizona State University (ASU)) were grown at 37°C in Luria-Bertani broth medium (LB, Fisher) under aerobic conditions. S*treptococcus mutans* UAB147 Serotype C (kindly provided by CIDV, ASU) and *Streptococcus pneumoniae* were grown at 37°C in Todd-Hewitt (TH, Fisher) broth medium with 5% horse blood under anaerobic conditions. *Escherichia coli* O157:H7 (ATCC) and *Burkholderia thailandensis* (ATCC) were cultured in Mueller-Hinton broth medium (MH, Fisher) while *Staphylococcus epidermidis* was cultured in TH broth medium.

### Peptide Microarray Preparation

Prior to spotting of the peptide library, polymer slides were prepared by: 1) Cleaning glass slides with Piranha solution (70∶30 v/v mixture of concentrated H_2_SO_4_ and 30% H_2_O_2_. WARNING: Piranha solution should be handled with caution and can detonate if mixed with significant quantities of oxidizable organic materials) for at least 1 hour at low rotation, followed by rinsing with H_2_O and drying. 2) Slides were silanized in 1% solution of 3-glycidoxypropyl-trimethoxysilane in anhydrous toluene for 30 minutes at 40°C, and washed with toluene (3 times). 3) Slides were cured for 40 minutes at 120°C. 4) Slides were coated with a solution of 6 mg/mL polyethylenimine in 10% ethanol for 1 hour at room temperature with agitation. 5) Slides were activated with sulfo-SMCC (Pierce Biotechnology, Rockford, IL, USA; Cat# 22622) to create a maleimide-activated surface. 6) The peptide library was then printed using a contact spotter on the activated slides. The maleimide-activated surface reacts with the sulfhydryl group on the peptide's terminal cysteine to orient the peptides on the surface.

### Microarray Assays

Before probing, the slides were treated with 90% trifluoroacetic acid (TFA) to remove non-immobilized peptides, blocking groups and probable organic impurities followed by dimethylfuran (DMF), ethanol and deionized water washing. Then, the slides were placed in a humidified chamber and blocked for 1 hour at room temperature with buffer [3% bovine serum albumin (BSA), 0.014% mercaptohexanol and 0.05% Tween-20 in 1×Tris buffered saline (TBS)].

Turbidity of overnight cultures was measured at OD_600_. The CFU/mL value was calculated according to the McFarland Equivalence Turbidity standard (Remel, R20421). Cell cultures were diluted to 8×10^8^ and washed 2 times with 1× phosphate buffered saline (PBS) buffer with 0.05% FBS (Fetal Bovine Serum, Invitrogen 10091-130). CTO staining solution was prepared by adding 500 µL of pre-warmed appropriate media with 10 µM CTO to one tube of washed cells and incubated in foil wrapped tube for 1 hour at 37°C at 250 rpm. Alexa Fluor 555 NHS ester (Invitrogen A32755) labeling solution was prepared by adding the 500 µL of 1×TBS/FBS with the content of one pre-packed dye vial dissolved in 10 µL DMSO to washed cells. The sample was incubated in a foil wrapped tube for 1 hour at room temperature with agitation. After staining/labeling, cells were washed with 1×TBS/FBS. The amount of dyes and incubation times vary for different pathogens and needed to be found experimentally.

After blocking the slides were washed with 1×TBS-T (1×30 inversion in a Coplin jar) and water (3×30 inversions in a Coplin jar). The slides were then dried by centrifugation at 1500 rpm for 2 minutes. Agilent hybridization chambers were used to ensure the interaction of the solution (10^8^ labeled cells in 1×TBS with 0.03% SodiµM azide, 3% BSA and 0.05% Tween 20 in total 450 µL) with the microarrays. To subtract false positive non-specific signals driven by dye binding, we conducted competitions with 20× excess of un-labeled cells. Each microarray assay was performed in triplicate. The slides were incubated for 1 hour at 37°C in the rotator (Agilent Technologies). Then slides were washed with 1×TBS-T (3×30 inversions in a Coplin jar) and water (3×30 inversions in a Coplin jar); the solution was changed each time. Finally, the slides were dried by centrifugation at 1500 rpms for 2 minutes and scanned.

### Microarray scanning and data analysis

Microarrays were scanned by using a Perkin-Elmer ProScanArray HT Microarray Scanner with the 488, 543 and 633 nm excitation lasers at 100% power and 70% photomultiplier tube gain. Detection was done at 570 nm for Cell Tracker Orange and AF555, at 508 nm for SYTO 9 and at 670 nm for DRAQ5. All scanned images were analyzed by using GenePix Pro 6.0 software (Axon Instruments, Union City, CA, USA). Upon careful visual inspection, bad spots were eliminated by flagging them absent. Median spot intensities were used in further analyses. Image-processed data were imported from GenePix for the following statistical analysis of microarray data to GeneSpring 7.2 (Agilent, Inc., Palo Alto, CA, USA). For correct analysis, each slide was normalized either to 50th percentile or by subtracting the local background from median intensity at each spot. Measurements of less than 0.01 were set to 0.01. The microarray profiles collected for each bacterial strain were compared by using scatter plots [25], [26]. Binding assay data were plotted versus competition assay data for each dye separately. Peptides that demonstrated at least 2 times higher intensity in binding assay were considered for subsequent analysis. Peptides with signals at both CTO and AF555 dyes (CTO+AF+) were considered as binders. Lytic peptides were selected as those with no signal in the CTO channel and signal in the AF555 channel (CTO-AF+). In order to distinguish specific peptide-binders and APs candidates the profiles of different strains were compared by Venn diagrams.

### Fluorescent Microscopy

For the microscopic detection of bacterial binding to the peptide microarray we printed custom slides with 10–20 peptides of interest. All procedures of microarray preparation and processing were the same as described above. After the last wash, 50 µL of 1×PBS was applied to the slides and spread under a cover slip. Binding was evaluated using fluorescent microscopy (Olympus BX61), at ×60 magnification with immersion oil with Cy3 excitation laser. Digital images were collected using factory-supplied software DP Controller 2.2.1.227 Olympus Corporation.

### Synbody Synthesis

Bivalent synbody was synthesized via a modified divergent solid phase peptide synthesis using Fmoc-Lys(ivDde)-OH as the scaffold using the protocols outlined in [19]. Synthesis was performed by removal of the Fmoc-protecting group followed by synthesis of peptide 1 on α-amino group of Lysine through stepwise addition of Fmoc amino acids. Upon completion of peptide 1 synthesis, the N-terminal Fmoc group was substituted with Boc group prior to deprotection of the Nε-(ivDde) protecting group. The stepwise assembly of peptide 2 was then accomplished at Nε-lysine position using stepwise addition of Fmoc-protected amino acids on the peptide synthesizer. The final protected synbody was treated with cleavage cocktail for 2 hours at room temperature and precipitated in cold diethyl ether. The solid was separated from diethyl ether by centrifugation and the top phase decanted off and pellet re-suspended with another addition of dry diethyl ether. The cooling and centrifugation processes were done in triplicate, as the construct was dried and dissolved in water for HPLC purification. Finally, the synbody was purified by HPLC and quality was analyzed by MALDI mass spectrometry.

### Minimum Inhibitory Concentration (MIC) and Kill Curve Kinetic Analysis

MIC determinations were conducted utilizing the broth microdilution assay according to the Clinical and Laboratory Standard [33] in Mueller-Hinton (MH; Fisher) broth medium at 35±2°C for EC, BS, PA, SA; in MH II (cation adjusted) with 5% horse blood for SM. CFU number controls and survivors control in bactericidal kinetics were conducted in MH agar for EC, BS, PA, SA; in MH II agar with 5% sheep blood for SM. For time kill curve studies, aliquots from each sample-treated well were removed at the indicated time point, diluted with 1× PBS, and serial dilutions of each sample were plated on agar plates. The plates were incubated overnight and bacterial colonies were counted. The results reported are the average of two experiments.

### Hemolytic assay

The protocol was adopted from Shin et al [34]. Female BALB/C mice were obtained from Charles River and housed in barrier isolation caging with food and water provided *ad libitum*. Mouse blood samples were collected via submandibular venipuncture using a 5.0 mm lancet (MEDIpoint, Inc., Mineola, NY) into heparinized tubes. All animal experiments were conducted following an animal use protocol (1099R) that was reviewed and approved by the Arizona State University Institutional Animal Care and Use Committee. Briefly, fresh mice erythrocytes were rinsed three times with PBS, centrifuged for 15 minutes at 900 g and resuspended in PBS. Samples (100 µL) of the suspension (4% in PBS, v/v) were plated in 96-well microtiter plates, after which 100 µL of the appropriate concentration peptide dissolved in PBS was added. Plates were incubated for 1 hour at 37°C and then centrifuged at 1000 g for 5 minutes. Aliqots (100 µL) of the supernatant were transferred to 96-well plates, where hemoglobin release was monitored using a microplate reader (Molecular Devices) by measuring the absorbance at 414 nm. Percent hemolysis was calculated by the following formula: % hemolysis = [(A414in the peptide solution – A414in PBS)/(A414in 0.1% Triton-X 100 – A414in PBS)] ×100. Zero and 100% hemolysis were determined in PBS and 0.1% Triton-X 100, respectively.

### Cytotoxicity assay

HEK293 (Human Embryonic Kidney cells were purchased from ATCC) (10^5^/mL cells) were seeded in individual wells of a microtitre plate and incubated for 24 hours at 37°C with 5% carbon dioxide in EMEM supplemented with 10% fetal bovine serum and Penicillin/Streptomycin. Cells were then challenged with synbody or peptides from 0.05 to 100 µM for either 24 or 48 hours. Cell viability was measured spectrophotometrically (450 nm) following the addition a peroxidase-conjugated anti-BrdU antibody, subsequent TMB degradation by peroxidase and stopping by acidic solution, as per the manufacturer's recommendations (Cell Proliferation ELISA, BrdU, Chemicon (Millipore)). Human tissue culture experiments were approved by Arizona State University Institutional Review Board.

### Co-culture of human embryonic kidney cells (HEK293) and *S. aureus*

Done accordingly to protocol [35] with modifications. Bacteria were grown on Mueller-Hinton (MH; Fisher) broth medium at 37°C for overnight. The bacteria were then harvested by centrifugation and the pellet suspended in Eagle Minimum Essential Medium (EMEM) supplemented with 10% fetal bovine serum without antibiotics. The bacterial density was adjusted to 10^8^ CFU/mL (stock). Serial dilutions of this concentration were prepared for further experiments.

HEK293 cells were cultured in complete EMEM (Minimum Essential Medium Eagle Media) supplemented with 10% fetal bovine serum and Penicillin/Streptomycin for 2 days in 75 cm^2^ cell culture flasks (Greiner) at 37°C in humidified atmosphere containing 5% CO_2_. For experiments, the cells were harvested through trypsin-EDTA treatment, seeded into 96 well tissue culture plates “Microtest” (Falcon) at concentration 4×10^5^ cells/mL. After 24 hours, the culture medium was replaced by either fresh EMEM without antibiotics (negative control) or *S. aureus* dilutions. To make the test conditions for the Synbody more restrictive, 1000× excess of minimal harmful concentration of *S. aureus* (1.25×10^6^ CFU/mL) was chosen for the set up of the co-culture system. Synbody and peptides RW and DR (25 µM) were added at 1 minute after starting the co-culture. After 24 hours of co-culture, the cell viability was determined visually and on the basis of a luminescence ATP detection assay “ATPlite” (Perkin Elmer). For the measurement of cellular ATP the cells were lysed (50 µL of mammalian cells lysis solution to 100 µL of cell suspension) for 5 minutes in an orbital shaker at 700 rpm. After lysis, a 50 µL of substrate solution containing Luciferase/Luciferin was added to react with released ATP. After 5 minutes incubation in an orbital shaker at 700 rpm, the emitted light was measured with a luminometer (Clarity, BioTek Instruments, Inc.). The ATP standard sample provided in ATPlite kit was diluted and measured to build a standard curve. Luminescence was converted to the cellular ATP content (nM) using the standard curve. The cellular viability under test conditions was expressed as percent of an untreated control (HEK293 cells).

## Supporting Information

Figure S1 **Peptide microarray surface chemistry.**
(TIF)

Figure S2 **Efficacy of functional assay for distinguishing of binding and lytic peptides directly on microarray.** AF555-NHS labeled EC, PA, BS, SM (x axis) plotted versus themselves in competition with 20× excess of non-labeled cells (y axis). Both axes show raw median fluorescent signal at 543 nm on a logarithmic scale. Green lines delimit the twofold change. Annotated dark dots are peptide-binders detected previously with CTO for each strain specifically. Peptides are classified “Binders” if repeated with AF (CTO+AF+) out of twofold compared to negative control. Other peptides in this area (red dots) have profile “CTO-AF+” and classified “Lytic”. Annotated peptides (black filled circles) within 2-fold change were ignored as CTO false positive signals. Note that some overlap in properties binder/lytic is possible when signal ratio AF/CTO is exceeding 1.5 for the peptide classified as binders and getting less than 2 for lytic peptides.
(TIF)

Figure S3 **Bacterial growth inhibition assay for synbody (red), peptides DR (green) and RW (black) for A)** ***S. epidermidis*** **B)** ***E. coli*** **O157:B7 C)** ***B. thailandensis*** **.**
(TIF)

Table S1 **MIC values for selected inhibitory peptides for each bacterium.**
(DOCX)

Table S2 **Kill curve kinetic studies of** ***S. aureus*** **binding peptide, inhibitory peptide, and synbody.**
(DOCX)
