# Supplementary figures and images for: A Technology for Developing Synbodies with Antibacterial Activity
Source: PLoS One. 2013 Jan 23;8(1):e54162. doi: 10.1371/journal.pone.0054162 (PMC3553175; doi:10.1371/journal.pone.0054162)

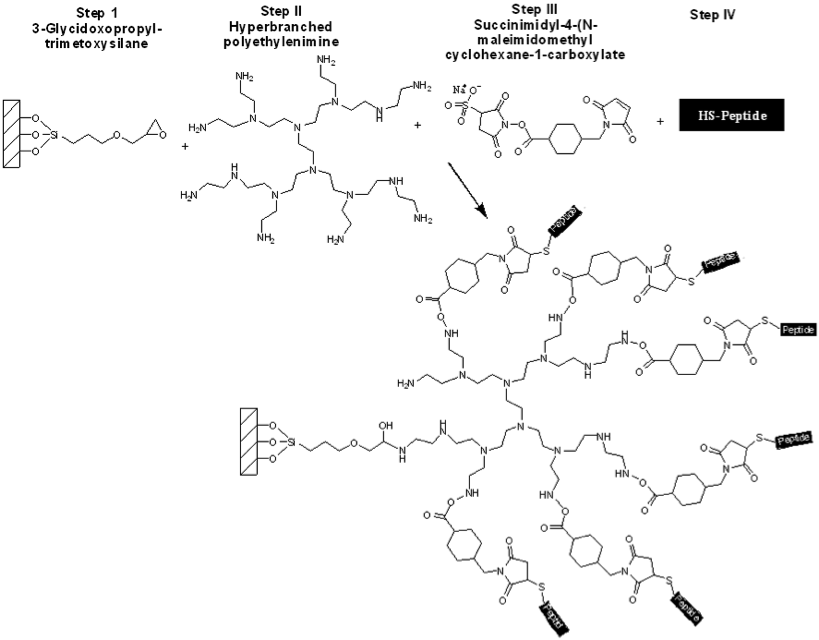

Supplement: Figure S1 — Peptide microarray surface chemistry. (TIF) [file pone.0054162.s001.tif]

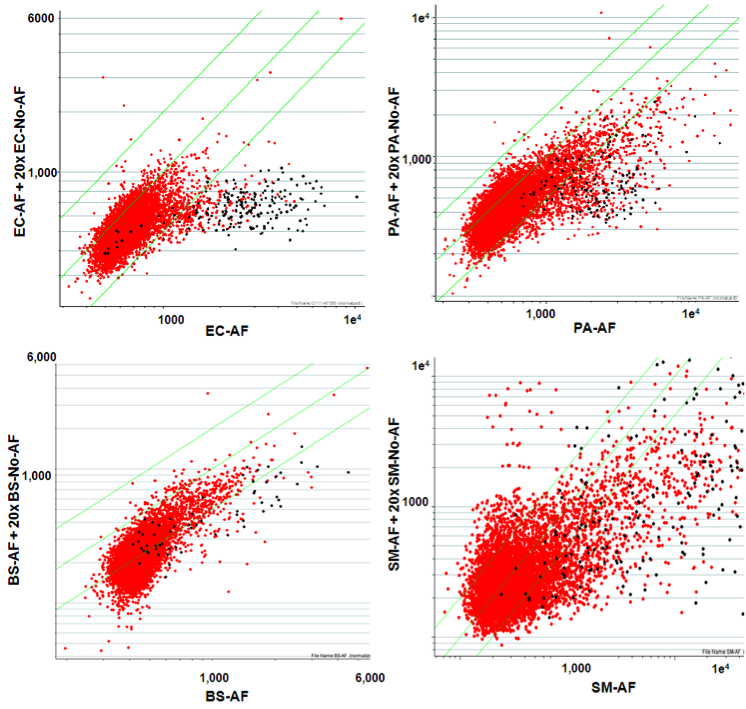

Supplement: Figure S2 — Efficacy of functional assay for distinguishing of binding and lytic peptides directly on microarray. AF555-NHS labeled EC, PA, BS, SM (x axis) plotted versus themselves in competition with 20× excess of non-labeled cells (y axis). Both axes show raw median fluorescent signal at 543 nm on a logarithmic scale. Green lines delimit the twofold change. Annotated dark dots are peptide-binders detected previously with CTO for each strain specifically. Peptides are classified “Binders” if repeated with AF (CTO+AF+) out of twofold compared to negative control. Other peptides in this area (red dots) have profile “CTO-AF+” and classified “Lytic”. Annotated peptides (black filled circles) within 2-fold change were ignored as CTO false positive signals. Note that some overlap in properties binder/lytic is possible when signal ratio AF/CTO is exceeding 1.5 for the peptide classified as binders and getting less than 2 for lytic peptides. (TIF) [file pone.0054162.s002.tif]

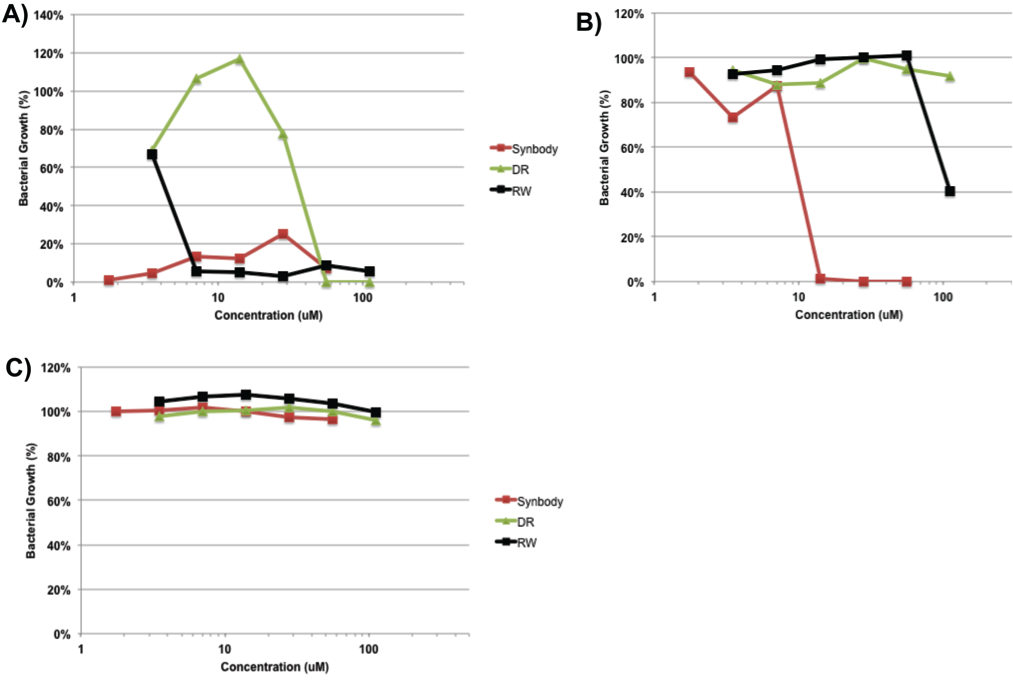

Supplement: Figure S3 — Bacterial growth inhibition assay for synbody (red), peptides DR (green) and RW (black) for A) S. epidermidis B) E. coli O157:B7 C) B. thailandensis . (TIF) [file pone.0054162.s003.tif]
