# Supplementary material for: A Technology for Developing Synbodies with Antibacterial Activity
Source: PLoS One. 2013 Jan 23;8(1):e54162. doi: 10.1371/journal.pone.0054162 (PMC3553175; doi:10.1371/journal.pone.0054162)
Supplement: Table S1 — MIC values for selected inhibitory peptides for each bacterium. (DOCX) [file pone.0054162.s004.docx]

**Supplementary Table S1.** MIC for selected inhibitory peptides for each bacterium.

|  | **Gram-positive Bacteria** | | | **Gram-Negative Bacteria** | |
| --- | --- | --- | --- | --- | --- |
|  | ***S. aureus*** | ***S. mutans*** | ***B. subtilis*** | ***E. coli* O111:B4** | ***P. aeruginosa*** |
| WKKKRKHRHKKHWHPWRGSC | 15 ± 1.1 μM | *N.I.* | 12 ± 1.8 μM | *N.I.* | *N.I.* |
| **RWRRHKHFKRPHRKHKRGSC** | **28 ± 1.5 μM** | ***N.I.*** | ***N.I.*** | ***N.I.*** | **27 ± 2.3 μM** |
| HPWWWHHKKRRHHKHKKGSC | 47 ± 3.2 μM | 16 ± 3.6 μM | *N.I.* | *N.I.* | 32 ± 2.9 μM |
| RHWRKPRKWHKKWPPHRGSC | 52 ± 4.8 μM | *N.I.* | 5 ± 0.3 μM | 92 ± 8.8 μM | 26 ± 3.7 μM |
| INAIVYKRRFQYVRPKIGSC | 60 ± 5.3 μM | *N.I.* | *N.I.* | 36 ± 3.3 μM | 97 ± 8.6 μM |
| WWHHKWFKHKKFWRHKFGSC | 78 ± 7.1 μM | *N.I.* | 80 ± 7.7 μM | *N.I.* | 25 ± 2.1 μM |
| YHNNPGFRVMQQNKLHHGSC | *N.I.* | *N.I.* | 26 ± 2.2 μM | *N.I.* | *N.I.* |
| HWKRRHKHKWPKRHPHKGSC | *N.I.* | *N.I.* | 10 ± 2.2 μM | 50 ± 5.8 μM | *N.I.* |
| HRPRKKFHKFPRKWRRHGSC | *N.I.* | *N.I.* | 8 ± 0.9 μM | 62 ± 7.2 μM | 34 ± 2.5 μM |
| WRHKPFFKWKPWKHFHWGSC | *N.I.* | *N.I.* | *N.I.* | 95 ± 9.1 μM | *N.I.* |
| WKFRHRHHRHHWHKKWKGSC | *N.I.* | *N.I.* | 4 ± 0.4 μM | *N.I.* | *N.I.* |
| KYKQNKITPINWILFIQGSC | *N.I.* | 55 ± 6.9 μM | *N.I.* | *N.I.* | *N.I.* |
| HKHWRRKPRHHRHWWWHGSC | *N.I.* | 95 ± 9.6 μM | *N.I.* | *N.I.* | *N.I.* |
| RIGTDMYKMQWQKTIGRGSC | *N.I.* | *N.I.* | 20 ± 3.6 μM | *N.I.* | *N.I.* |
| VLKHHRVKAFKFWHEYIGSC | *N.I.* | *N.I.* | 32 ± 4.5 μM | *N.I.* | *N.I.* |
| **DRIFHKMQHKPYKIKKRGSC** | ***N.I.*** | ***N.I.*** | ***N.I.*** | ***N.I.*** | ***N.I.*** |
