# Supplementary material for: A Technology for Developing Synbodies with Antibacterial Activity
Source: PLoS One. 2013 Jan 23;8(1):e54162. doi: 10.1371/journal.pone.0054162 (PMC3553175; doi:10.1371/journal.pone.0054162)
Supplement: Table S2 — Kill curve kinetic studies of S. aureus binding peptide, inhibitory peptide, and synbody. (DOCX) [file pone.0054162.s005.docx]

**Table S2.** Kill curve kinetic studies of *S. aureus* binding peptide, inhibitory peptide, and synbody.

|  |  | **CFU/mL at indicated time (hr)** | | | | | | |
| --- | --- | --- | --- | --- | --- | --- | --- | --- |
| **Compound** | **Conc.** | **0** | **0.5** | **2** | **4** | **6** | **9** | **24** |
| Kanamycin | Cells Only | 4.8x10^7^ | 1.5x10^8^ | 2.3x10^8^ | 8.8x10^8^ | 3.1x10^9^ | 4.6x10^9^ | 6.8x10^9^ |
|  | MIC | 4.8x10^7^ | 1.6x10^6^ | 3.2x10^7^ | 1.6x10^6^ | 1^a^ | 8.3x10^6^ | 1^a^ |
|  | 2 x MIC | 4.8x10^7^ | 1.6x10^6^ | 5.8x10^6^ | 1^a^ | 1^a^ | 1^a^ | 1^a^ |
|  | 4 x MIC | 4.8x10^7^ | 4.7x10^6^ | 1^a^ | 1^a^ | 1^a^ | 1^a^ | 1^a^ |
|  |  |  |  |  |  |  |  |  |
| Peptide DR | Cells Only | 4.8x10^7^ | 1.5x10^8^ | 2.3x10^8^ | 8.8x10^8^ | 3.1x10^9^ | 4.6x10^9^ | 6.8x10^9^ |
|  | 25 µM | 4.8x10^7^ | 1.2x10^8^ | 1.6x10^8^ | 8.8x10^8^ | 1.7x10^9^ | 3.2x10^9^ | 5.3x10^9^ |
|  | 50 µM | 4.8x10^7^ | 3.2x10^8^ | 5.3x10^8^ | 5.4x10^8^ | 2.4x10^9^ | 2.2x10^9^ | 3.9x10^9^ |
|  | 100 µM | 4.8x10^7^ | 7.0x10^7^ | 1.2x10^9^ | 6.0x10^8^ | 2.1x10^9^ | 2.0x10^9^ | 5.6x10^9^ |
|  |  |  |  |  |  |  |  |  |
| Peptide RW | Cells Only | 4.8x10^7^ | 1.5x10^8^ | 2.3x10^8^ | 8.8x10^8^ | 3.1x10^9^ | 4.6x10^9^ | 6.8x10^9^ |
|  | 25 µM | 4.8x10^7^ | 4.5x10^7^ | 1.5x10^8^ | 1.0x10^9^ | 1.7x10^9^ | 2.4x10^9^ | 4.4x10^9^ |
|  | 50 µM | 4.8x10^7^ | 3.2x10^7^ | 1.0x10^8^ | 7.2x10^8^ | 2.0x10^9^ | 2.4x10^9^ | 3.8x10^9^ |
|  | 100 µM | 4.8x10^7^ | 7.2x10^6^ | 5.1x10^7^ | 2.5x10^8^ | 1.4x10^9^ | 3.4x10^9^ | 2.7x10^9^ |
|  |  |  |  |  |  |  |  |  |
| Synbody | Cells Only | 4.8x10^7^ | 1.5x10^8^ | 2.3x10^8^ | 8.8x10^8^ | 3.1x10^9^ | 4.6x10^9^ | 6.8x10^9^ |
|  | 12.5 µM | 4.8x10^7^ | 8.1x10^7^ | 1.3x10^8^ | 3.9x10^8^ | 2.2x10^9^ | 2.7x10^9^ | 5.4x10^9^ |
|  | 25 µM | 4.8x10^7^ | 8.7x10^7^ | 2.0x10^7^ | 2.9x10^8^ | 1.6x10^9^ | 2.7x10^9^ | 3.7x10^9^ |
|  | 50 µM | 4.8x10^7^ | 1.8x10^7^ | 4.9x10^7^ | 1.8x10^8^ | 1.1x10^9^ | 2.6x10^9^ | 3.3x10^9^ |

1^a^ = indicates bactericidal result (>3 log_10_ reduction)
